# A Nomogram for Predicting Patent Foramen Ovale-Related Stroke Recurrence

**DOI:** 10.3389/fneur.2022.903789

**Published:** 2022-06-09

**Authors:** Zhuonan Wu, Chuanjing Zhang, Nan Liu, Wenqing Xie, Jinjin Yang, Hangyuan Guo, Jufang Chi

**Affiliations:** ^1^Shaoxing University School of Medicine, Shaoxing, China; ^2^Ningbo University School of Medicine, Ningbo, China; ^3^Zhejiang Chinese Medical University of Medicine, Hangzhou, China; ^4^Zhejiang University School of Medicine, Hangzhou, China; ^5^Department of Cardiology, The First Affiliated Hospital of Shaoxing University (Shaoxing People's Hospital), Shaoxing, China

**Keywords:** PFO-related stroke, nomogram, prognosis, forward stepwise Cox regression, LASSO regression

## Abstract

**Background:**

The high prevalence of patent foramen ovale (PFO) in cryptogenic stroke suggested a stroke-causing role for PFO. As risk factors for recurrence of such stroke are not recognized, clinicians cannot sufficiently identify, treat, and follow-up high-risk patients. Therefore, this study aimed to establish a prediction model for PFO-related stroke recurrence.

**Methods:**

This study included 392 patients with PFO-related stroke in a training set and 164 patients with PFO-related stroke in an independent validation set. In the training set, independent risk factors for recurrence identified using forward stepwise Cox regression were included in nomogram 1, and those identified using least absolute shrinkage and selection operator(LASSO)regression were included in nomogram 2. Nomogram performance and discrimination were assessed using the concordance index (C-index), area under the curve (AUC), calibration curve, and decision curve analyses (DCA). The results were also validated in the validation set.

**Results:**

Nomogram 1 was based on homocysteine (Hcy), high-sensitivity C-reactive protein (hsCRP), and albumin (ALB), and nomogram 2 was based on age, diabetes, hypertension, right-to-left shunt, ALB, prealbumin, hsCRP, and Hcy. The C-index of nomogram 1 was 0.861, which was not significantly different from that of nomogram 2 (0.893). The 2- and 5-year AUCs of nomogram 1 were 0.863 and 0.777, respectively. In the validation set, nomogram 1 still had good discrimination (C-index, 0.862; 2-year AUC, 0.839; 5-year AUC, 0.990). The calibration curve showed good homogeneity between the prediction by nomogram 1 and the actual observation. DCA demonstrated that nomogram 1 was clinically useful. Moreover, patients were successfully divided into two distinct risk groups (low and high risk) for recurrence rate by nomogram 1.

**Conclusions:**

Nomogram 1, based on Hcy, hsCRP, and ALB levels, provided a more clinically realistic prognostic prediction for patients with PFO-related stroke. This model could help patients with PFO-related stroke to facilitate personalized prognostic evaluations.

## Introduction

Approximately 30–40% of ischemic strokes are known as “cryptogenic strokes” because they have no identified cause (1). In the last century, epidemiological surveys have found a significantly higher prevalence of patent foramen ovale (PFO) in the cryptogenic stroke population than in the normal population, suggesting a stroke-causing role for PFO (2). The main mechanism of PFO-related stroke is currently believed to be paradoxical embolism, in which a thrombus from the vein (especially the deep vein of the lower extremity) travels through the venous system to the right atrium and through the PFO to the left atrium, which is known as a right-to-left shunt (RLS), then the thrombus followed by blood flow into cerebral arteries, leading to stroke. In 2017, the publication of the results of the RESPECT long-term follow-up, the CLOSE trial, and the Gore-REDUCE trial verified that PFO can cause stroke and strongly demonstrated that PFO closure is superior to drug treatment alone in reducing the recurrence of PFO-related stroke (3–5). Consequently, scholars formally proposed the concept of “PFO-related stroke” in 2020 (6). Although PFO closure resulted in a relative (65%) reduction in the risk of stroke recurrence compared with antiplatelet treatment alone (7), the relatively low absolute recurrence rate of PFO-related stroke itself and the lack of accurate assessment of patient prognosis allow that a large number of patients remain on conservative drug therapy and some even remain untreated. Despite the absence of data on the long-term risk of stroke recurrence (>10 years) in patients with PFO-related stroke receiving drug therapy, more than 50% of patients were followed up for 5 years or longer. In these trials (3), the Kaplan-Meier curves for drug treatment did not show a decrease in the rate of stroke recurrence over time. In some studies, the annual recurrence rate of PFO-related stroke remained as high as approximately 4.8%, and this figure even increased to 8.2% in some patients with PFO-related stroke (8). Therefore, it is essential to identify the population at a high risk of PFO-related stroke recurrence, which is also a current clinical limitation. There is no consensus regarding risk factors for PFO-related stroke recurrence or clinically available biological markers. Although some studies have identified larger PFO size, larger RLS, atrial septal aneurysm (ASA), and high homocysteine (Hcy) levels as risk factors for PFO-related stroke recurrence, there is still a lack of consistent understanding and integration of risk scores for each independent risk factor.

## Methods

The data supporting the findings of this study are available from the corresponding author upon reasonable request.

### Study Design and Population

This is a two-way cohort study in which we assessed, included, informed, and followed up patients with cryptogenic stroke according to the Trial of Org 10,172 in acute stroke treatment (TOAST) who visited the stroke center of Shaoxing People's Hospital, Zhejiang Province between January 2016 and July 2021. The main inclusion criteria were as follows: (1) age 18–70 years and (2) recent cerebral infarct lesion detected using cranial magnetic resonance imaging (MRI) during hospitalization and PFO detected using contrast transesophageal echocardiography with or without ASA. The major exclusion criteria were as follows: (1) risk of paradoxical embolism score <4 (9), indicating that stroke has less correlation with PFO; (2) atherosclerosis or lumen stenosis >50% or occlusion of intracranial and cervical vessels; (3) suspected cardiogenic embolism (atrial fibrillation, moderate to severe mitral stenosis, etc.); (4) small vessel occlusion disease (lacunar infarction), namely, presence of small deep infarcts (<1.5 cm in diameter) or typical clinical lacunar syndrome; (5) other definite causes such as arterial dissection, vasculitis, neoplasms, hypercoagulable state, and drug use; and (6) presence of other acute phase infections. Ischemic stroke was defined as acute focal neurological symptoms that lasted for more than 24 h or symptoms that lasted for < 24 h but with new cerebral infarct lesions detected using cranial MRI. All patients who met the inclusion criteria were diagnosed as having PFO-related stroke by two or more neurologists and were treated with standardized therapy, including drugs (antiplatelet) alone or closure therapy.

All participants provided informed consent before recruitment. The study protocol was approved by the Ethics Committee of Shaoxing People's Hospital according to the Declaration of Helsinki.

### Data Collection

We collected and recorded patients' clinical data from the medical record system, including (1) general data such as sex, age, and admission time; (2) past and personal history, including hypertension, diabetes, stroke history, and smoking history; (3) biological markers such as albumin (ALB), prealbumin (PA), total cholesterol (CHOL), apolipoprotein A1 (ApoA1), apolipoprotein B (ApoB), high-density lipoprotein (HDL), low-density lipoprotein (LDL), high-sensitivity C-reactive protein (hsCRP), Hcy, and D-dimer; and (4) others such as PFO size, RLS, ECG, echocardiography findings, and cranial MRI.

### Follow-Up and End Point

Follow-up through telephone and notification of follow-up visits were conducted 1, 3, 6, and 12 months after the index stroke and every 12 months until 5 years. The primary end point was recurrent ischemic stroke that was defined as the onset of a new neurological deficit that met the ischemic stroke criteria or sudden worsening of a previous deficit. The occurrence of recurrent ischemic stroke was confirmed based on clinical presentation, medical records, imaging findings, or other available data at each follow-up visit and was diagnosed by neurologists as being associated with PFO.

### Statistical Analysis

Continuous variables were transformed into classification variables using the X-Tile software to choose the best cutoff values so that the model was more objective and simple. The classification thresholds for each index were as follows: age, 55 years; PFO size, 1.5 mm; RLS, 5 microbubbles; ALB, 38.95 mmol/L; CHOL, 5.69 mmol/L; HDL, 1.22 mmol/L; LDL, 1.94 mmol/L; ApoA1, 1.09 mmol/L; ApoB, 0.99 mmol/L; PA, 216.45 mmol/L; hsCRP, 2.64 mmol/L; and Hcy, 12.4 mmol/L. The computer randomized all patients into training and validation sets at a ratio of 7:3. Comparisons between the groups were performed using the chi-square test and *t*-test.

Based on the training set, forward stepwise Cox regression was used to screen variables to establish nomogram 1, and least absolute shrinkage and selection operator (LASSO) regression was used to establish nomogram 2. Receiver operating characteristic curve (ROC) analysis and Harrell's concordance index (C-index) were used to assess the discrimination of the model. Bootstrap calibration curves with 1,000 resamples were used to assess the calibration of the nomograms. Decision curve analysis (DCA) was used to assess the clinical benefit of alternative models and was applied to the nomograms by quantifying the net benefit at different threshold probabilities. Curves for the treat-all patient protocol (representing the highest clinical cost) and the no-treatment protocol (representing no clinical benefit) were plotted as two references in the DCA. In the nomograms, points were assigned by drawing a line upward from the corresponding values to the “points” line. The sum of these points, plotted on the “total points” line, corresponded to the prediction of 2- and 5-year stroke recurrence-free rates in patients with PFO-related stroke. Patients were subsequently divided into low- and high-risk groups based on the median value of the total points. Kaplan-Meier curves were then plotted, and the log-rank test was used to compare risk stratification. C-index takes values in the range of 0.5–1.0, where 0.5–0.7 indicates random chance, >0.7 indicates reasonable estimation, and 1.0 indicates perfect estimation. The 45° line of the calibration chart indicates ideal perfect prediction, the solid blue line indicates the predictive performance of the nomograms, and the distance between the blue line and the 45° line represents the predictive accuracy of the nomogram. Finally, we compared the indicators of nomograms 1 and 2 and selected a suitable nomogram for further internal verification.

Statistical analyses were performed using the SPSS (26.0 IBM, Armonk, NY, USA) and R (version 4.1.2) software. The R version 4.1.2 for the “glmnet” package was used for LASSO regression; the “Rms” “survival” packages for nomograms, calibration curves, and C-index calculation; the “timeROC” package for time-dependent ROC curves; and “dcurves” for DCA. Statistical significance was set at *p-*values <0.05.

## Results

### Basic Characteristics

Overall, 556 patients were included in this study. The baseline characteristics were not significantly different between the training and validation sets, with the exception of ApoA1 (Table 1).

**Table 1:** Characteristics of patients in the training and validation sets.

**Variables**	**Training set (*n* = 392)**	**Validation set (*n* = 164)**	***P*-value**
Sex (*n*%)			
Female	149 (38.0%)	71 (43.3%)	
Male	243 (62.0%)	93 (56.7%)	0.245
Age (years)			
>55	249 (63.5%)	100 (61.0%)	
< 55	143 (36.5%)	64 (39.0%)	0.571
Hypertension (*n*%)			
With	236 (60.2%)	87 (53.0%)	
Without	156 (39.8%)	77 (47.0%)	0.119
Diabetes (*n*%)			
With	73 (18.6%)	29 (17.7%)	
Without	319 (81.4%)	135 (82.3%)	0.794
Smoking (*n*%)			
With	133 (33.9%)	53 (32.3%)	
Without	259 (66.1%)	111 (67.7%)	0.713
Stroke history (*n*%)			
With	35 (8.9%)	9 (5.5%)	
Without	357 (91.1%)	155 (94.5%)	0.171
RoPE			
>7	82 (20.9%)	46 (28.0%)	
<7	310 (79.1%)	118 (72.0%)	0.069
PFO size (mm)			
>1.5	270 (68.9%)	118 (72.0%)	
<1.5	122 (31.1%)	46 (28.0%)	0.472
RLS (microbubbles)			
>5	188 (48.0%)	85 (51.8%)	
<5	204 (52.0%)	79 (48.2%)	0.405
ALB (g/L)			
>38.95	282 (71.9%)	121 (73.8%)	
<38.95	110 (28.1%)	43 (26.2%)	0.657
CHOL (mmol/L)			
>5.69	42 (10.7%)	19 (11.6%)	
<5.69	350 (89.3%)	145 (88.4%)	0.764
HDL (mmol/L)			
>1.22	153 (39.0%)	62 (37.8%)	
<1.22	239 (61.0%)	102 (62.2%)	0.787
LDL (mmol/L)			
>1.94	351 (89.5%)	147 (89.6%)	
<1.94	41 (10.5%)	17 (10.4%)	0.974
ApoA1 (g/L)			
>1.09	303 (77.3%)	113 (68.9%)	
<1.09	89 (22.7%)	51 (31.1%)	0.038
ApoB (g/L)			
>0.99	128 (32.7%)	55 (33.5%)	
<0.99	264 (67.3%)	109 (66.5%)	0.840
PA (mg/L)			
>216.45	344 (87.8%)	142 (86.6%)	0.705
<216.45	48 (12.2%)	22 (13.4%)	
hsCRP (mg/L)			
>2.64	92 (23.5%)	31 (18.9%)	0.237
<2.64	300 (76.5%)	133 (81.1%)	
Hcy (μmol/L)			
>12.4	112 (28.6%)	37 (22.6%)	0.145
<12.4	280 (71.4%)	127 (77.4%)	
Recurrence (*n*%)			
With	23 (5.9%)	8 (4.9%)	0.125
without	369 (94.1%)	156 (95.1%)	
Follow–up time (months)	19.3 ± 14.47	19.4 ± 14.28	0.880

*PFO,patent foramen ovale; RLS, right–to–left shunting; ALB, albumin; PA, prealbumin; CHOL, total cholesterol; ApoA1, apolipoprotein A1; ApoB, apolipoprotein B; HDL, high–density lipoprotein; LDL, low–density lipoprotein; hsCRP, high–sensitivity C–reactive protein; Hcy, homocysteine*.

### Establishing the Nomograms

**Figure 1:**
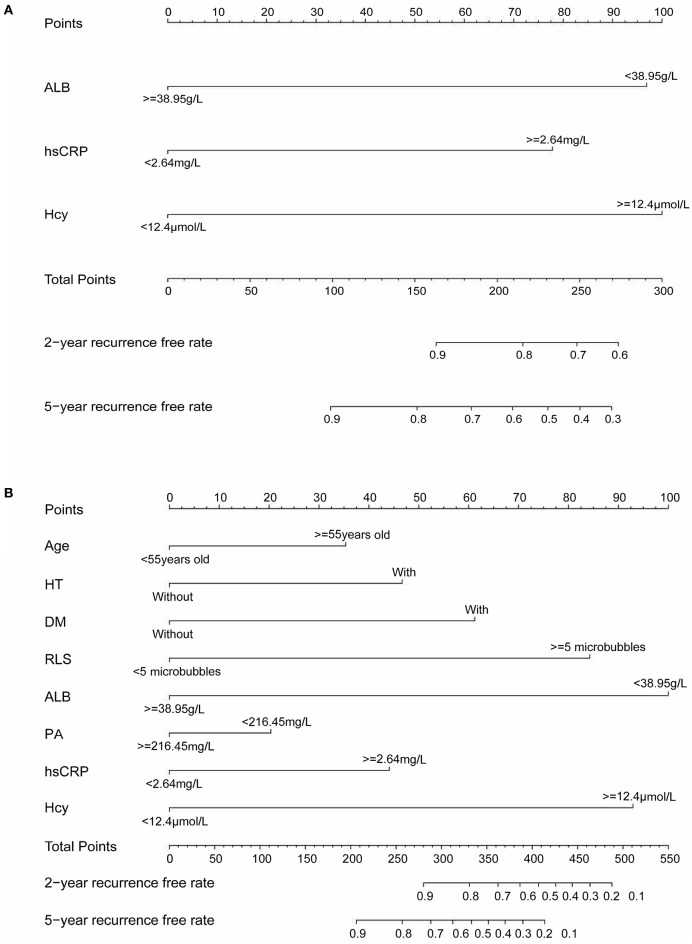
**(A)** Nomogram 1 conducted by Cox regression, including Hcy, hsCRP, and ALB in patients with PFO-related stroke. **(B)** Nomogram 2 conducted by LASSO regression, including age, diabetes, hypertension, RLS, ALB, PA, hsCRP, and Hcy in patients with PFO-related stroke. Nomogram 1 and nomogram 2 are used to calculate the 2- and 5-year stroke recurrence-free rate. For an individual patient, each variable corresponds to a “Points” in the first row. The “Total Points” are summed up by all points and indicated in the third row from the bottom. Drawing a vertical line from “Total Points” to the first and second rows from the bottom will respectively show the 2- and 5-year recurrence-free rate.

**Figure 2:**
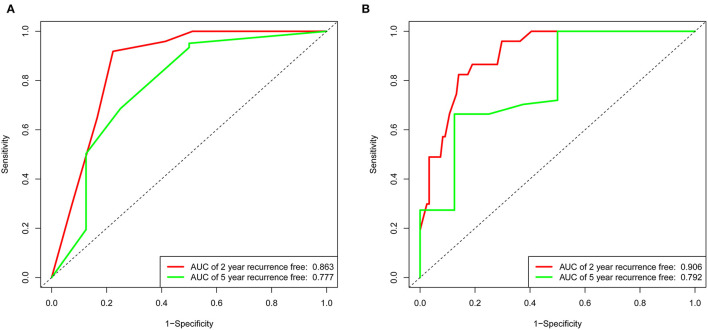
Receiver operating characteristic curve of the nomogram 1 and nomogram 2 in the training set. **(A)** The red line represents 2-year recurrence-free in nomogram 1. The green line represents 5-year recurrence-free rate in nomogram 1. **(B)** The red line represents 2-year recurrence-free rate in nomogram 2. The green line represents 5-year recurrence-free rate in nomogram 2.

The variables associated with PFO-related stroke recurrence in the univariate Cox analysis are shown in Table 2. Forward stepwise Cox regression analysis (Table 2) identified ALB (>38.95 mmol/L vs. <38.95 mmol/L; hazard ratio [HR], 0.406 [95% CI, 0.191–0.864]; *p* = 0.019), hsCRP (>2.64 vs. <2.64 mmol/L; HR, 3.721 [95% CI, 1.755–7.887]; *p* = 0.001), and Hcy (>12.4 vs. <12.4 mmol/L; HR, 5.782 [95% CI, 2.453–13.630]; *p* < 0.001) as independent risk factors of PFO-related stroke recurrence. Nomogram 1 (Figure 1A) was constructed using these significant predictors in the forward stepwise Cox regression analysis. The C-index for nomogram 1 was 0.861, and the 2- and 5-year AUCs were 0.863 and 0.777, respectively. The 2- and 5-year ROC curves are shown in Figure 2A.

**Table 2:** Univariate and multivariate Cox analysis of the training set.

**Variables**	**Univariate analysis** **HR (95 % CI)**	***P*-value**	**Multivariate analysis** **HR (95 % CI)**	***P*-value**
Sex				
Female vs. Male	0.289 (0.110–0.754)	0.011		
Age (years)				
>55 vs. <55	3.588 (1.375–9.364)	0.009		
Hypertension				
With vs. Without	2.176 (0.972–4.868)	0.059		
Diabetes mellitus				
With vs. Without	1.910 (0.848–4.300)	0.118		
Smoking				
With vs. Without	2.654 (1.302–5.411)	0.007		
Stroke history				
With vs. Without	1.520 (0.523–4.313)	0.450		
RoPE				
>7 vs. <7	0.286 (0.087–0.944)	0.040		
PFO size (mm)				
>1.5 vs. <1.5	0.386 (0.189–0.792)	0.009		
RLS (microbubbles)				
>5 vs. <5	1.789 (0.839–3.814)	0.132		
ALB (g/L)				
>38.95 vs. <38.95	0.241 (0.117–0.498)	<0.001	0.406 (0.191–0.864)	0.019
CHOL (mmol/L)				
>5.69 vs. <5.69	0.814 (0.192–3.450)	0.780		
HDL (mmol/L)				
>1.22 vs. <1.22	0.312 (0.108–0.902)	0.031		
LDL (mmol/L)				
>1.94 vs. <1.94	0.501 (0.214–1.171)	0.110		
ApoA1 (g/L)				
>1.09 vs. <1.09	0.511 (0.245–1.064)	0.073		
ApoB (g/L)				
>0.99 vs. <0.99	0.680 (0.276–1.678)	0.403		
PA (mg/L)				
>216.45 vs. <216.45	0.267 (0.126–0.563)	0.001		
hsCRP (mg/L)				
>2.64 vs. <2.64	5.623 (2.724–11.604)	<0.001	3.721 (1.755–7.887)	0.001
Hcy (μmol/L)				
>12.4 vs. <12.4	7.586 (3.250–17.709)	<0.001	5.782 (2.453–13.630)	<0.001

**Figure 3:**
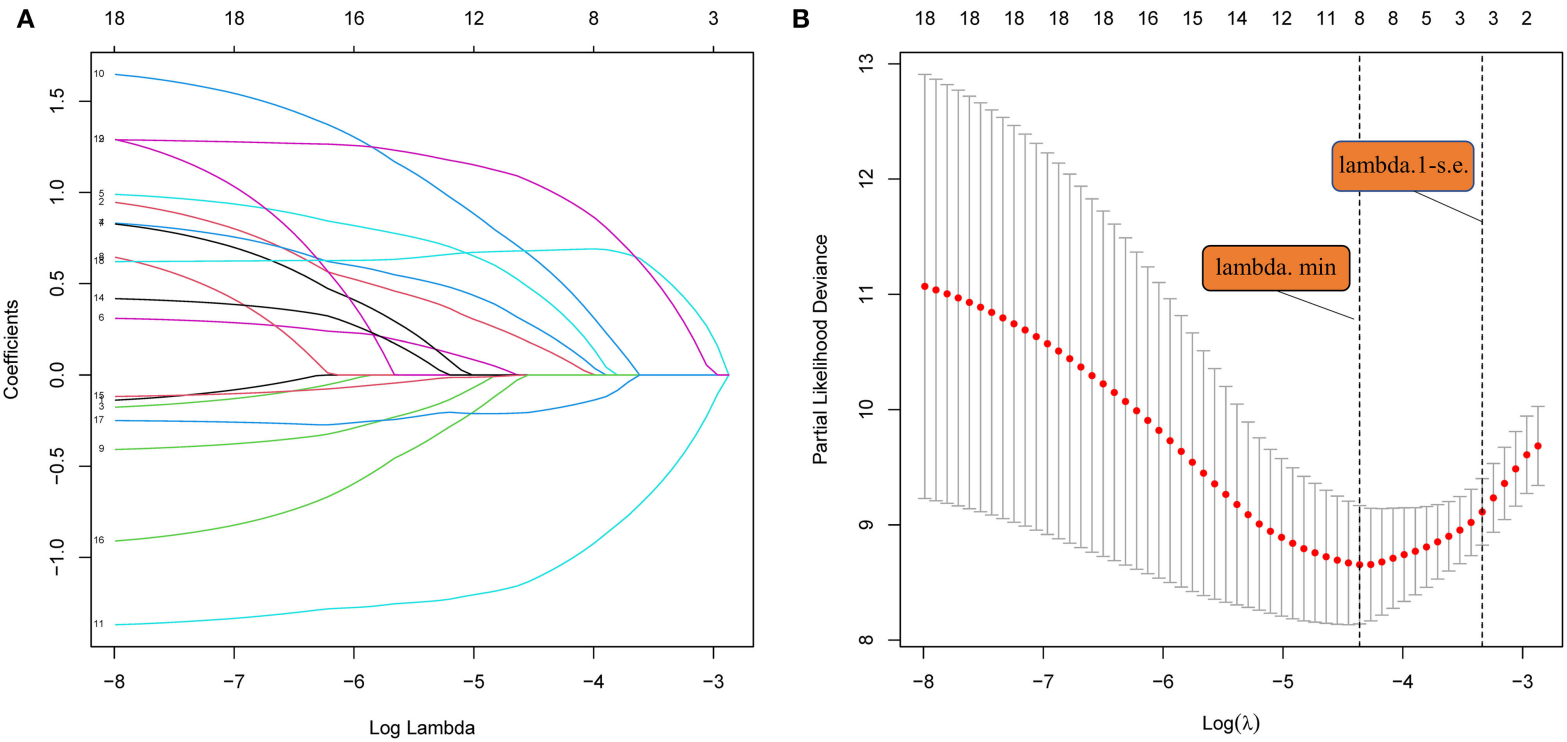
**(A)** LASSO coefficient profiles of the 18 risk factors. **(B)** Risk factors selected using LASSO regression analysis. The two dotted lines were drawn at the optimal scores by minimum criteria and 1-s.e. criteria (at minimum criteria, including age, diabetes, hypertension, RLS, ALB, PA, hsCRP, and Hcy at 1-s.e. criteria, including ALB, hsCRP, and Hcy).

LASSO regression (Figure 3) showed an association between age, diabetes, hypertension, RLS, ALB, PB, hsCRP, Hcy, and PFO-related stroke recurrence at minimum values. Further disciplinary regression was performed to consider the 1-s.e. criteria ALB, hsCRP, and Hcy as independent risk factors for prognosis in patients with PFO-related stroke. As the predictors screened by the 1-s.e. criterion as the same as the forward stepwise Cox regression, nomogram 2 was established with minimum values (Figure 1B). The C-index for nomogram 2 was 0.893, and the 2- and 5-year AUCs were 0.906 and 0.792, respectively. The 2- and 5-year ROC curves are shown in Figure 2B.

**Figure 4:**
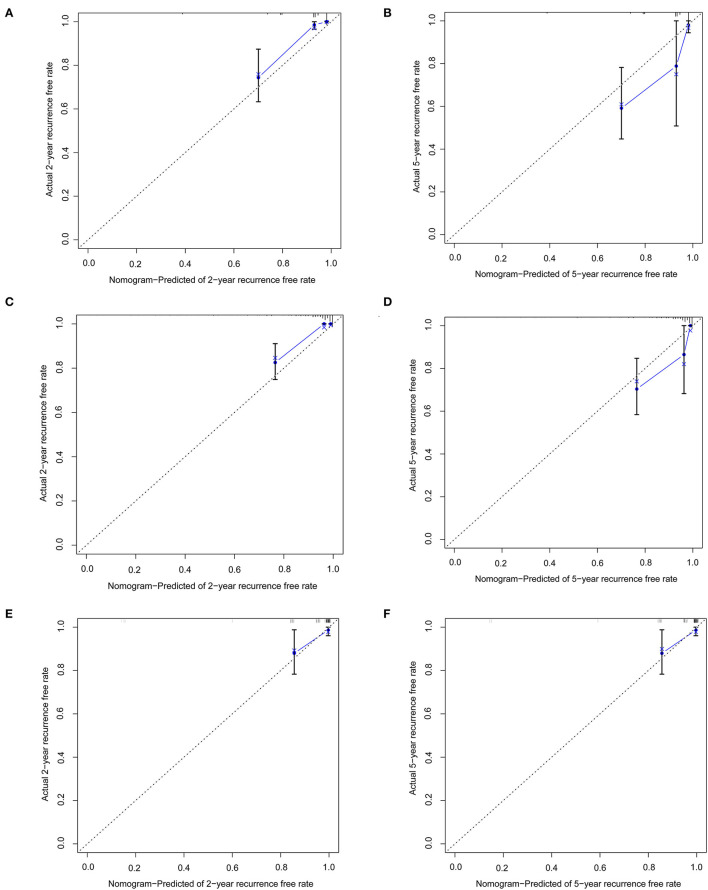
Calibration curves represent the difference between the actual prediction (blue line) and the ideal perfect prediction (45° line). **(A)** 2-year recurrence free rate in the training set of nomogram 1. **(B)** 5-year recurrence-free rate in the training set of nomogram 1. **(C)** 2-year recurrence-free rate in the training set of nomogram 2. **(D)** 5-year recurrence-free rate in the training set of nomogram 2. **(E)** 2-year recurrence-free rate in the validation set of nomogram 1. **(F)** 5-year recurrence-free rate in the validation set of nomogram 1.

**Figure 5:**
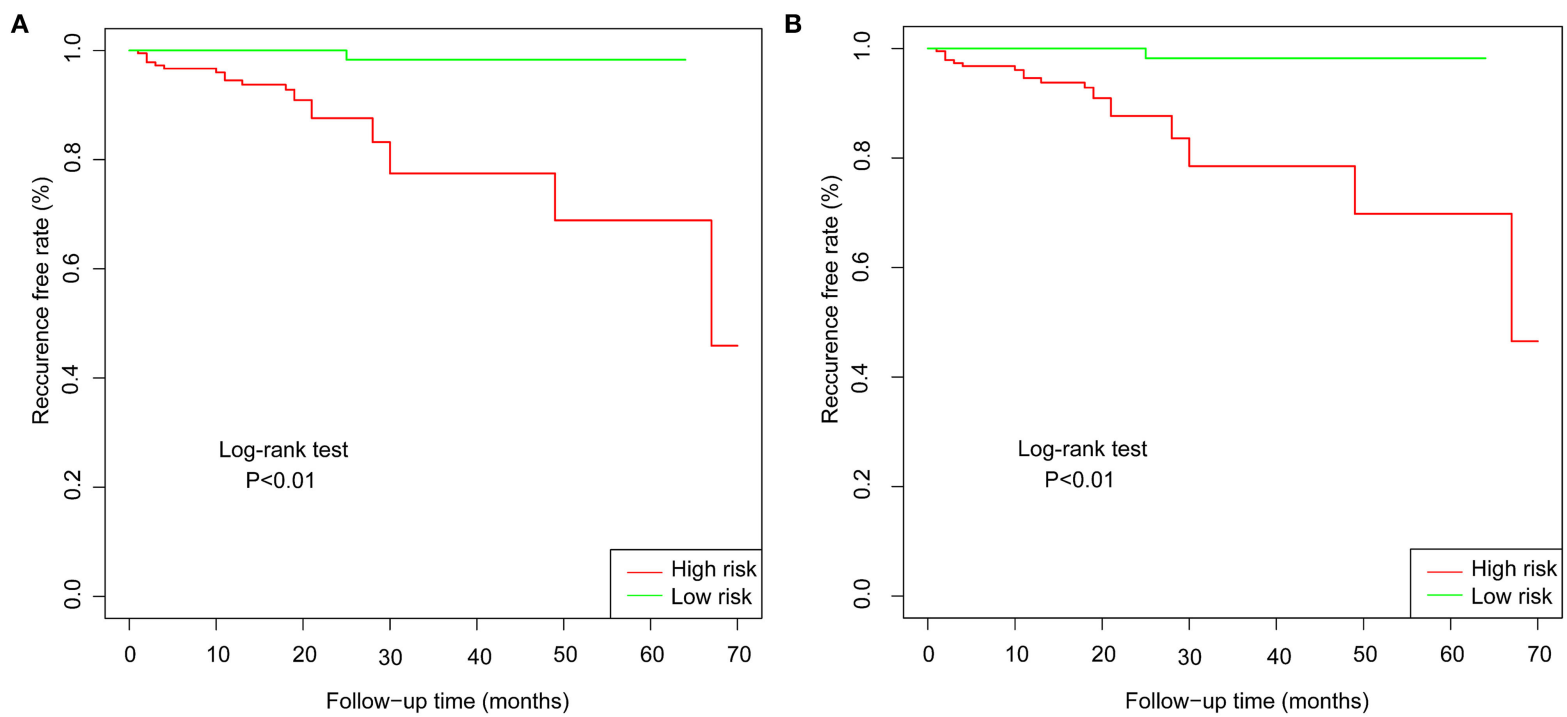
**(A)** Kaplan-Meier curves for low-risk and high-risk populations stratified by nomogram 1 in the training set. **(B)** Kaplan-Meier curves for low-risk and high-risk populations stratified by nomogram 2 in the training set.

**Figure 6:**
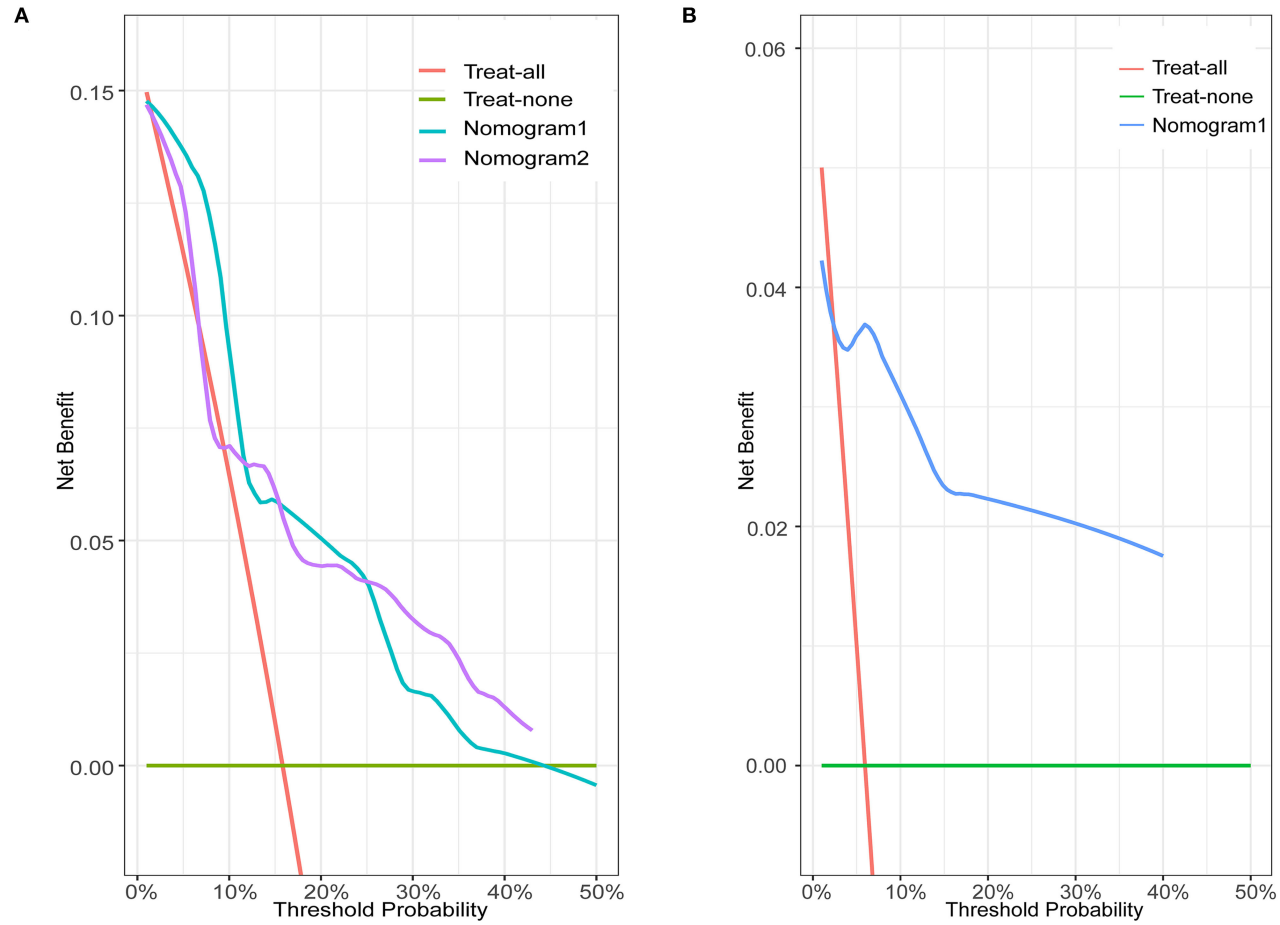
Decision curve analysis for nomograms in the training and validation sets. Green horizontal line: treat-none patient protocol (representing no clinical benefit). Red line: treat-all patient protocol (representing the highest clinical cost). **(A)** Blue line: training set of nomogram 1. Purple line: training set of nomogram 2. **(B)** Blue line: validation set of nomogram 1.

In the training set of both nomograms, the C-index and the 2- and 5-year AUCs were all >0.7, illustrating good discrimination between the two prediction models. The 2- and 5-year calibration curves (Figure 4) showed the best consistency between the predicted and actual risks in these two nomograms. The Kaplan-Meier curves (Figure 5) showed a large difference between both risk groups, with nomograms 1 and 2 performing well in their ability to identify high-risk patients. DCA (Figure 6A) determined whether the model provided greater net benefits. Nomogram 1 ensured greater net benefits than the treat-all and no-treatment protocols when the risk threshold was between 0.02 and 0.43; however, nomogram 2 only ensured larger returns at risk thresholds of 0.02–0.07 and 0.09–0.46. DCA performances of nomograms 1 and 2 were comparable in the intersection area of the risk thresholds.

Compared to nomogram 2, nomogram 1 had significantly fewer model variables with similar predictive efficacy and high clinical practical value. Therefore, this study only provides further internal validation of nomogram 1.

### Internal Validation

**Figure 7:**
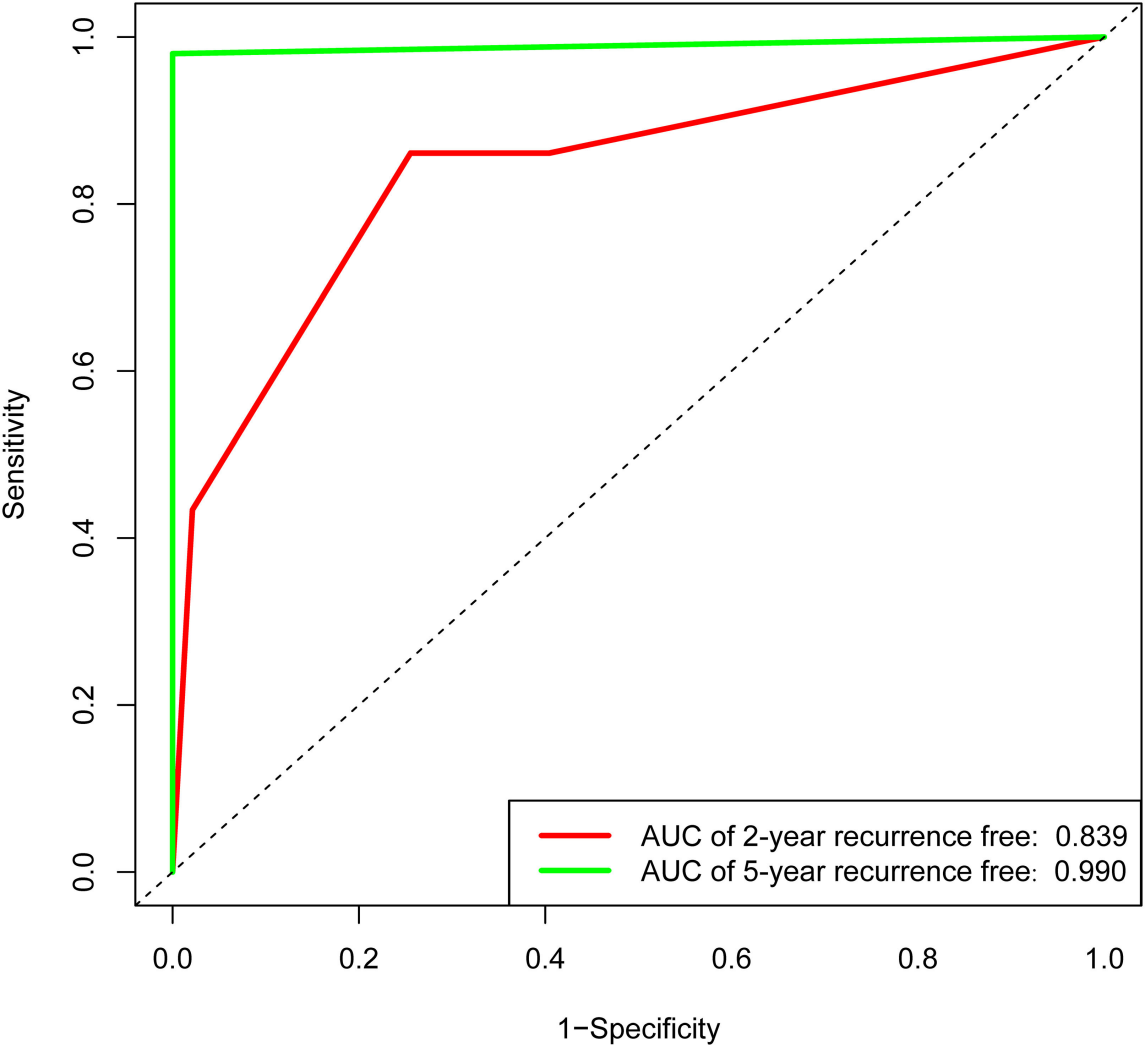
Receiver operating characteristic curve of the nomogram 1 in the validation set. The red line represents 2-year recurrence-free rate. The green line represents 5-year recurrence-free rate.

**Figure 8:**
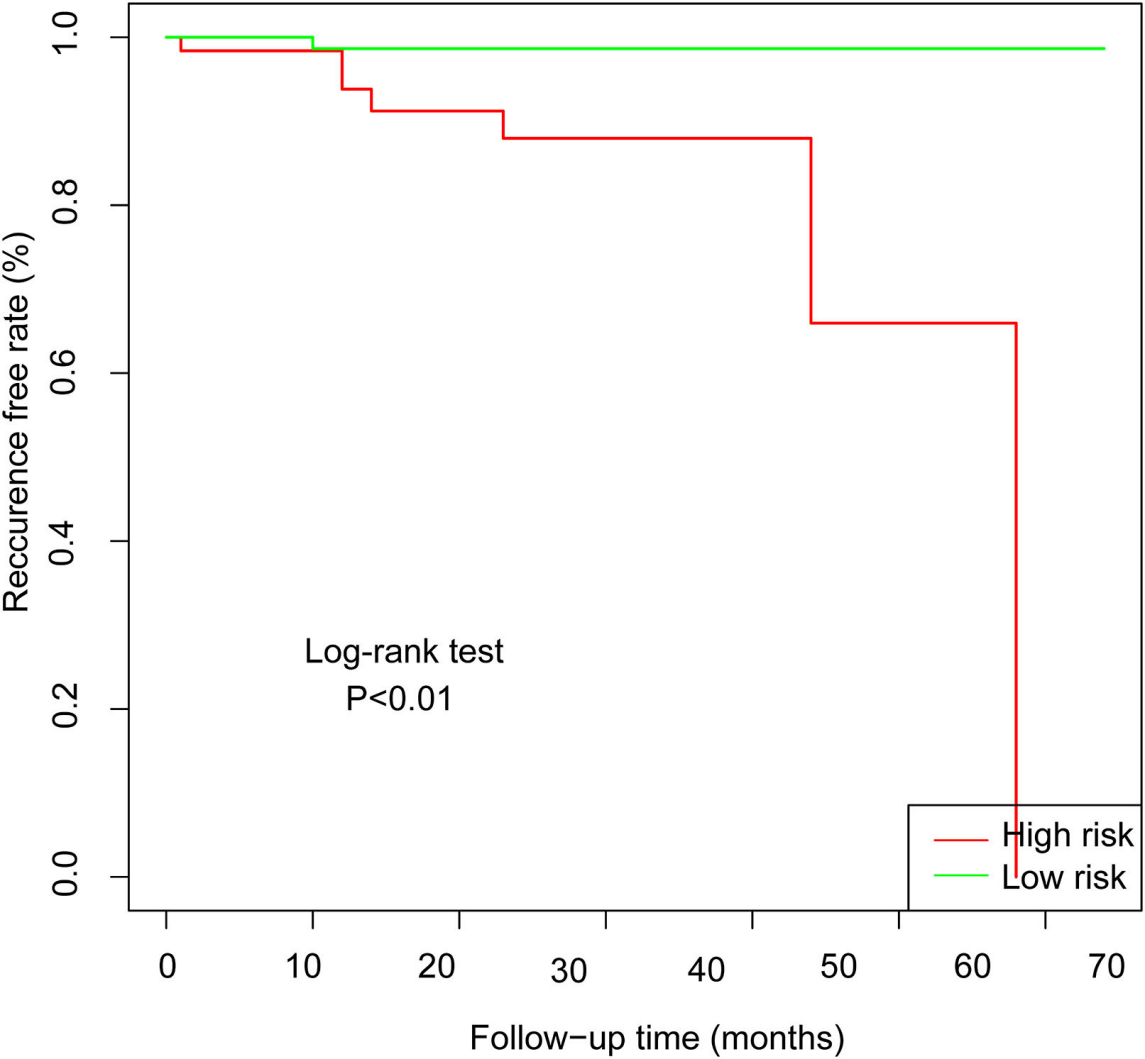
Kaplan-Meier curves for low-risk and high-risk populations stratified by nomogram 1 in the validation set.

In the validation set of nomogram 1, the C-index was 0.862, and the 2- and 5-year AUCs were 0.839 and 0.990, respectively. The 2- and 5-year ROC curves are shown in Figure 7; calibration curves are shown in Figure 4; and the Kaplan-Meier curves are shown in Figure 8. Regarding clinical practicability, larger net benefits were obtained for almost all risk thresholds (Figure 6B). For the above data, we obtained better results in the validation set than in the training set, which illustrates the usefulness of nomogram 1 in clinical decision-making.

## Discussion

To date, no prediction models for PFO-related stroke recurrence have been found. It is necessary to create a prediction model to predict stroke recurrence. Therefore, we created nomograms using two statistical methods, rigorously evaluated and internally validated the performance of the nomograms, and finally selected nomogram 1, which was more practical. Based on the model's risk stratification, clinicians can select more aggressive treatment strategies and closely follow-up high-risk patients to benefit them at more frequent assessments after discharge.

Most previous studies were limited to stepwise Cox regression for the screening of risk factors, which is not conducive to small sample sizes and multi-index models. LASSO regression analysis performs penalized regression on all variable coefficients so that relatively unimportant independent variable coefficients become zero, thereby excluding them from modeling and obtaining a more refined model (10). The biggest difference between LASSO regression and traditional stepwise Cox regression is that the former can process all independent variables simultaneously instead of in a stepwise manner. This improvement allows much greater stability in the modeling. Therefore, LASSO regression can not only solve the problem of overfitting but also effectively extract useful features, which is more suitable for decision-making with more clinical indicators. In this study, our goal was to select a model with as few variable features as possible that would be more suitable for clinical practice. LASSO regression at 1-s.e. criteria screened the same model variables as forward stepwise Cox regression (Hcy, hsCRP, and ALB), indicating that the results had high confidence. Nomogram 1 was constructed using these significant predictors. At the same time, to avoid missing the prediction model with higher discrimination, accuracy, and stability, we also established nomogram 2 with eight variables selected using LASSO regression at minimum values. In the training set, the C-index (0.861 vs. 0.893), 2-year AUC (0.863 vs. 0.906), 5-year AUC (0.777 vs. 0.990), 2- and 5-year calibration curves (Figure 4), and Kaplan-Meier curves of nomograms 1 and 2 (Figure 5) showed less difference. Moreover, the DCA performance of nomogram 2 was slightly inferior (Figure 6A). After comprehensive consideration, we chose nomogram 1 based on the three variables of Hcy, hsCRP, and ALB. We also achieved better results in the validation set of nomogram 1.

The Hcy level was the variable with the highest predictive score in the nomogram 1. Hcy has been reported to be an independent risk factor for the development of cerebral infarction (especially large atherosclerotic and cardiogenic embolic types) (11, 12). In the field of stroke recurrence, some studies have also revealed its risk in recent years; however, few articles have reported its role in stroke recurrence owing to PFO. It is notable that our previous study suggested that Hcy is a risk factor for recurrence of this type of stroke (13), but definite conclusions could not be made because of a small sample size. This study expanded the sample size and used multiple statistical methods, all of which suggested that Hcy contributes to PFO-related stroke recurrence, thereby increasing the credibility of this conclusion.

The mechanism by which Hcy causes PFO-related stroke recurrence may be related to pulmonary circulation and anatomical features of the foramen ovale. It is currently hypothesized that the presence of RLS allows harmful substances to accumulate in the peripheral circulation, thereby increasing the risk of stroke recurrence. The kidney is the major organ for Hcy clearance, but enzymes involved in Hcy metabolism in the kidney, such as methylenetetrahydrofolate reductase and 5-methyltetrahydrofolate-Hcy methyltransferase, as well as Hcy uptake and transport systems, are also expressed in the pulmonary circulation (14, 15). In addition, in pulmonary diseases, such as chronic obstructive pulmonary disease and pulmonary hypertension, Hcy is elevated, indicating that the lungs are involved in the metabolism and absorption of Hcy (16). A study by Deng et al. (17) directly compared the metabolomics of left atrial blood before and after PFO closure and found that Hcy was significantly lower in the left atrium after closure than before closure, and long-term follow-up results also showed that PFO closure enabled patients to maintain lower levels of Hcy in venous blood over time compared to before PFO closure. This suggests that Hcy is the main substance that evades the clearance effect of pulmonary circulation *via* the RLS mechanism of PFO. In addition, it has been well demonstrated that Hcy has a procoagulant effect and promotes venous thrombosis (18, 19), which is consistent with our previous study showing a higher recurrence rate in patients with PFO-related stroke with high Hcy levels (13). What's more, Hcy may directly or indirectly contribute to the development of atrial cardiopathy, particularly silent episodes of atrial fibrillation, which could lead to stroke. The mechanisms may be related to electrical and structural remodeling, namely, (1) Hcy directly affects ion channels (especially potassium channels) in atrial myocytes and (2) Hcy can cause biochemical damage, affect the structure and function of cellular proteins, alter redox status, and ultimately lead to pathological changes, such as endothelial dysfunction, vascular smooth muscle cell proliferation and changes in the property of interstitial fibers with extracellular matrix remodeling (20, 21). The abovementioned metabolomic study by Deng et al. (17) found that there were other differential substances in addition to Hcy, but this has not been subsequently reported and may be a direction for future research on mechanisms and risk factors.

The hsCRP used in this prediction model is a marker of inflammation. The Framingham study reported that inflammatory markers (such as C-reactive protein, interleukin-6, and fibrinogen) were associated with the risk of ischemic stroke recurrence and transient ischemic attack (22, 23). However, hsCRP has previously been considered an unreliable predictor of ischemic stroke recurrence. First, the elevated hsCRP is likely to be associated with stroke severity (brain tissue lesion size, neurological deficits, etc.), which itself is associated with prognosis (24, 25). This means that hsCRP is likely to be a marker only and is not involved in the mechanism that leads to stroke recurrence. Second, as hsCRP is affected by many factors, it fluctuates greatly and is unstable. However, after adjusting for potential confounding factors (such as stroke severity, infarct volume, and cardiovascular risk factors), many studies (26, 27) in recent years have concluded that hsCRP is still independently associated with stroke recurrence. An increasing number of studies have considered hsCRP not only as an inflammation marker but also as a biomarker of cardiovascular events, which can predict the prognosis of patients with stroke (28–31). This study focused on the effects of continuous chronic inflammation on stroke recurrence in patients with PFO-related stroke. Therefore, we excluded excessive hsCRP in the acute phase, obtained mean hsCRP values during follow-up after discharge, and found hsCRP to be a risk factor for stroke recurrence.

The mechanism by which hsCRP contributes to PFO-related stroke recurrence may be related to its pro-atrial cardiopathy and procoagulant effects (32). It has been suggested that hsCRP may play a role in promoting atrial cardiopathy in cryptogenic strokes. Studies showed that inflammation may alter the conduction properties of atrial myocytes through atrial remodeling mechanisms (including activation of atrial fibroblasts, gap junction damage caused by changes in connexins, and abnormal intracellular calcium handling), contribute to the development and persistence of atrial fibrillation, and finally lead to the prothrombotic state in this group of patients (33). Furthermore, inflammation could increase the risk of thromboembolism in the atrial cardiopathy group even without atrial fibrillation (34). The relationship between hsCRP and the coagulation system may rely on the concept of “immunothrombosis” with activating each other, ultimately leading to thrombosis. For example, neutrophils in inflammation can lead to a highly prothrombotic state by releasing neutrophil extracellular traps (35, 36). Some pro-inflammatory cytokines can induce endothelial cells and monocytes to produce tissue factors that activate coagulation (37, 38). In turn, thrombin, FXa, and FVIIa produced after the activation of the coagulation system have pro-inflammatory characteristics, which can stress the endothelium and produce pro-inflammatory cytokines, thereby causing further inflammation (39). Therefore, inflammation may have a significant role in PFO-related stroke, but the exact mechanism is unclear.

In this prediction model, ALB was significantly associated with stroke recurrence. A study in northern Manhattan (40) followed 2,986 individuals without stroke for 12 years and found that a lower serum ALB level was a predictor of cryptogenic stroke incidence after adjustment for cardiovascular risk factors, body mass index, and inflammation. Subsequently, a large number of observational studies strongly support the correlation between serum ALB and stroke prognosis (41–44). This mechanism may be related to the antioxidant effects of ALB. ALB is the most abundant antioxidant in whole blood carrying nitric oxide, bilirubin, etc. (45), which can prevent venous thrombosis and reduce the occurrence of PFO-related stroke. Therefore, hypoalbuminemia may be a risk factor that cannot be ignored and is easily treated.

All the above indicate that the pathogenesis of PFO-related stroke may not be single and is related to various stroke types under the current standard. So, the stroke caused by PFO possibly should not be limited to cryptogenic stroke.

Previous studies have suggested that anatomical features of PFO, such as PFO size and RLS, are associated with the first occurrence of PFO-related stroke. However, in this study, forward stepwise Cox regression did not show that the relevant anatomical structures were related to the recurrence of such strokes. Probably because the included patients had already experienced PFO-related stroke, meaning the foramen ovale in this population is susceptible to pass embolus. Therefore, for stroke recurrence, susceptibility to venous thrombosis is more important than the anatomical structure of the PFO. Some special structures of PFO, such as eustachian valve, long tunnel, and ASA, were not included in the variables for analysis as these were rarely present in the included population. In addition, the aim of this study was to help clinicians assess the risk of recurrence based on various clinical indicators for patients with PFO-related stroke at the first visit prior to human intervention. Therefore, the patient's treatment was not included as a variable.

### Limitations

Although our nomograms performed well, several limitations must be addressed. First, the limited number of patients with PFO-related stroke, all from the same hospital, and the lack of external validation may have created bias in assessing the predictive value of these markers. Second, as this is a two-way cohort study to predict future stroke recurrence, our results still need to be confirmed with a larger sample size and longer follow-up period. Third, we only included clinical indicators commonly used in this unit; further biomarkers were not detected. Perhaps other biological markers would be of greater value.

## Data Availability Statement

The raw data supporting the conclusions of this article will be made available by the authors, without undue reservation.

## Ethics Statement

The studies involving human participants were reviewed and approved by the Ethics Committee of Shaoxing People's Hospital. The patients/participants provided their written informed consent to participate in this study.

## Author Contributions

ZW, CZ, and JC: paper design and conception. ZW and CZ: manuscript writing. ZW, CZ, NL, and WX: manuscript revision and editing. CZ, JY, and HG: tables and figures. All authors agree to be accountable for the content of the work. All authors contributed to the article and approved the submitted version.

## Funding

This study was supported by the Zhejiang Province 325 Talent Program [6001383] and General Project of Zhejiang Health Science, Technology Plan [2021KY360].

## Conflict of Interest

The authors declare that the research was conducted in the absence of any commercial or financial relationships that could be construed as a potential conflict of interest.

## Publisher's Note

All claims expressed in this article are solely those of the authors and do not necessarily represent those of their affiliated organizations, or those of the publisher, the editors and the reviewers. Any product that may be evaluated in this article, or claim that may be made by its manufacturer, is not guaranteed or endorsed by the publisher.
